# Dietary Iron Concentration May Influence Aging Process by Altering Oxidative Stress in Tissues of Adult Rats

**DOI:** 10.1371/journal.pone.0061058

**Published:** 2013-04-12

**Authors:** Lorena Fernandes Arruda, Sandra Fernandes Arruda, Natália Aboudib Campos, Fernando Fortes de Valencia, Egle Machado de Almeida Siqueira

**Affiliations:** 1 Health Sciences Faculty, Campus Universitário Darcy Ribeiro, Universidade de Brasília, Brasília, DF, Brazil; 2 Nutrition Department of Health Sciences Faculty, Campus Universitário Darcy Ribeiro, Universidade de Brasília, Brasília, DF, Brazil; 3 Cell Biology Department of Biological Sciences Institute, Campus Universitário Darcy Ribeiro, Universidade de Brasília, Brasília, DF, Brazil; Temple University, United States of America

## Abstract

Iron is an essential element. However, in its free form, iron participates in redox-reactions, leading to the production of free radicals that increase oxidative stress and the risk of damaging processes. Living organisms have an efficient mechanism that regulates iron absorption according to their iron content to protect against oxidative damage. The effects of restricted and enriched-iron diets on oxidative stress and aging biomarkers were investigated. Adult Wistar rats were fed diets containing 10, 35 or 350 mg/kg iron (adult restricted-iron, adult control-iron and adult enriched-iron groups, respectively) for 78 days. Rats aged two months were included as a young control group. Young control group showed higher hemoglobin and hematocrit values, lower levels of iron and lower levels of MDA or carbonyl in the major studied tissues than the adult control group. Restricted-iron diet reduced iron concentrations in skeletal muscle and oxidative damage in the majority of tissues and also increased weight loss. Enriched-iron diet increased hematocrit values, serum iron, gamma-glutamyl transferase, iron concentrations and oxidative stress in the majority of tissues. As expected, young rats showed higher mRNA levels of heart and hepatic L-Ferritin (Ftl) and kidneys SMP30 as well as lower mRNA levels of hepatic Hamp and interleukin-1 beta (Il1b) and also lower levels of liver protein ferritin. Restricted-iron adult rats showed an increase in heart Ftl mRNA and the enriched-iron adult rats showed an increase in liver nuclear factor erythroid derived 2 like 2 (Nfe2l2) and Il1b mRNAs and in gut divalent metal transporter-1 mRNA (Slc11a2) relative to the control adult group. These results suggest that iron supplementation in adult rats may accelerate aging process by increasing oxidative stress while iron restriction may retards it. However, iron restriction may also impair other physiological processes that are not associated with aging.

## Introduction

Oxygen radicals produced endogenously during cellular respiration promote the accumulation of oxidative damage in tissues, resulting in cellular aging [1]. The balance between the production of reactive oxygen species (ROS) and antioxidant defenses determines the oxidative state of the body. An increase in the production of ROS or a reduction in the antioxidant capacity of the body induces progressive oxidative damage in tissues, leading to the organ dysfunction that characterizes aging [1], [2], [3]. According to the theory of the role of free radicals in aging, as postulated by Denham Harman in the mid-1950s, animals with high metabolic rates have reduced lifespans.

Due to its chemical versatility, iron was most likely a critical catalyst for the formation of complex organic compounds in the primitive environment and then became more commonly used in modern biological systems [4]. Although iron is an essential nutrient for health and is involved in several biological processes, such as erythropoiesis, oxidative metabolism, cellular immune responses and the regulation of cell growth and differentiation [5], free iron can potentially become toxic [6], [7]. Superoxide radicals (O_2_^•−^), which are produced via the electron transport chain, may reduce ferric ions to ferrous ions, which in turn may react with hydrogen peroxide (H_2_O_2_) by the Fenton reaction. This reaction produces the hydroxyl radical (OH^•^), which is one of the most reactive ROS [6]. Iron overload favors the availability of redox-active labile iron in the tissues and thus the increase of free radical production and oxidative damage to lipids, proteins and DNA that may lead to organ dysfunction [7]. The potentially deleterious effects of iron, in addition to their essential role in animal biochemical processes, drove the evolution of a molecular system of proteins that transport iron through membranes such as divalent metal transporter 1 (DMT-1); transferrin receptor; ferroportin; ferritin, which stores iron in tissues and transferrin, which provides iron mobility through the blood circulation in a safe and available way for the metabolic needs of biological systems [8], [9], [10]. Cellular iron content post-transcriptionally regulates the levels of DMT-1, ferritin, transferrin receptor and ferroportin by the iron regulatory proteins (IRP1 or IRP2) and the iron responsive element (IRE) system (IRP/IRE), which are untranslated regions of mRNA located in the 5′ or 3′ end [3], [11], while hepcidin, a hormone primarily synthesized in the liver, regulates systemic iron homeostasis by adjusting iron absorption and mobilization in accordance with physiological requirements [8], [10]. However, despite these efficient molecular mechanisms that control iron homeostasis in the body, tissue iron levels typically increase with age, presumably due to a failure in the regulation of iron absorption in enterocytes [12], [13].

Several studies have suggested a positive relationship between the loss of iron homeostasis, the accumulation of oxidative damage and cellular aging [14], [15], [16]. Some authors suggest the use of iron chelation as an alternative therapy for diseases associated with aging and oxidative stress [17], [18], [19]. Iron deficiency during the reproductive period in women maybe the principal factor responsible for their longevity compared to men, as iron has been referred to as the malignant spirit of premature aging [18].

Thus, considering that iron accumulation in tissues may be the causative factor for the accumulated oxidative damage in senescent cells [5], this work was based on the hypothesis that chronic oral iron supplementation is harmful and may contribute to aging. To test this hypothesis, we investigated the effects of the consumption of enriched and restricted chronic iron diets on molecular biomarkers of oxidation in tissues and on the expression of gene aging markers in rats. The specific activity of the enzymes catalase, glutathione reductase, glutathione peroxidase, glutathione-S-transferase and oxidized NADPH and the levels of lipid peroxidation and protein carbonylation in tissues were analyzed as tissue oxidative status biomarkers. Gene level expression of senescence marker protein (SMP30), ferritin light chain (Ftl) and the peptide hormone hepcidin (Hamp) were analyzed by quantitative RT-PCR as markers of aging [12], [20], [21]. The mRNA levels of nuclear factor erythroid derived 2 like 2 (Nfe2l2) and cAMP-responsive element-binding protein H (Creb3l3), both hepatic transcriptional factors which are responsible for the oxidative stress in liver were analyzed [22], [23], [24], [25], [26]. The transcript level of Slc11a2 gene that synthesize the divalent metal transporter – 1 (DMT-1), which is responsible by iron uptake was also analyzed in gut [27]. The transcript level of interleukin – 1 beta (Il1b) was analyzed as an inflammatory response marker [28].

## Materials and Methods

### Animals

Twenty adult male Wistar rats (approximately 15 months), provided by Bioagri's Laboratory located in Planaltina, Distrito Federal, Brazil, were divided into three groups of five, seven and eight animals and housed individually in stainless steel cages at a temperature of 22±2°C under a 12 h light/dark cycle. The rats had free access to water and food during the dark cycle. The rats were treated for 78 days (12 weeks) with one of the following diets: the adult control group received a rodent diet, the AIN-93M diet, containing 35 mg/kg iron [29]; the adult restricted-iron group (ARI) received the AIN-93M diet containing 10 mg/kg iron; and the adult enriched-iron group (AEI) was fed with the AIN-93M diet containing 350 mg/kg iron. The iron dose in the enriched-iron diet was estimated from the “tolerable upper limit intake” for humans (70 kg, adult), adjusting the value proportionally to the recommendation for rats. The iron dose in the restricted-iron diet corresponds to the inherent contamination of the diet ingredients, which is estimated at 30% of the recommendation described for AIN-93M. Six rats aged 2 months were included as a young control group one week before sacrifice. After the treatment period, rats were sacrificed by cervical dislocation, blood samples was collected by cardiac puncture into two tubes with and without EDTA 7.0% (21 µl/ml blood) and the organs—liver, spleen, heart, kidney, skeletal muscle (lower limb) and gut (1-cm length of small intestine distal to the pylorus and 1-cm proximal to the ileocecal valve was excised and the lumen was rinsed with saline)—were excised, washed in a cold 0.9% NaCl solution, rapidly frozen in liquid nitrogen and stored at −70°C to determine body iron status and mRNA levels of cAMP responsive element binding protein H (Creb3l3), hepcidin antimicrobial peptide (Hamp), ferritin light polypeptide (Ftl), senescence marker protein-30 (SMP30), nuclear factor erythroid derived 2 like 2 (Nfe2l2), interleukin – 1 beta (Il1b) and the iron divalent metal transporter- 1 (Slc11a2). This protocol was approved by the Ethics Committee for the Use of Animals of the Institute of Biological Sciences, University of Brasilia, Brazil as UnBDOC No. 100.199/2009.

### Weight record and food intake

The animals were weighed weekly during the treatment. The consumption of the diet was measured daily by comparing differences in weight between the amount of food offered and left.

### Hematological parameters

Hematological parameters were measured by the Sabin® Laboratory (Distrito Federal, Brazil). Red blood cells, hemoglobin and hematocrit were analyzed using an automated hematological analyzer. C-reactive protein concentration (CRP) was measured using an immunoturbidimetric method, which is based on the agglutination of latex particles coated with anti-CRP antibodies when they are mixed with serum containing CRP. Gamma glutamyl transferase (GGT) activity was determined by kinetic colorimetric assay. The iron parameters for serum iron, transferrin saturation and transferrin concentration were determined using the colorimetric ferrozine-based assay (Goodwin modified).

### Tissue iron determination

The concentration of iron in tissues was determined using the method described by Baranowska et al. (1995) [30]. Briefly, samples of liver, spleen, heart, kidney, skeletal muscle and gut were digested with 5 mL concentrated HNO_3_ (Sigma Aldrich Co., St. Louis, MO, USA) and 2.5 mL H_2_SO_4_ (Sigma Aldrich Co., St. Louis, MO, USA) in a Provecto Analítica Microwave System (DGT 100 Plus, Jundiai, São Paulo, Brazil, 2003). After digestion, the samples were resuspended in 0.1 mol/L nitric acid to a final volume of 25 mL. The concentration of iron in the samples was determined by inductively coupled plasma atomic emission spectrometry (ICP – AES/Spectro, Kleve, Germany) using a 238 nm line. A calibration curve was obtained for the range from 0 to 10 ppm solution of Fe-Merck Titrisol (Merck, Darmstadt, Germany) to calculate the concentrations of iron in the tissues.

### RNA extraction and reverse transcription-polymerase chain reaction analysis (qRT-PCR)

The extraction of total RNA from heart, gut, kidney and liver was performed using Trizol reagent (Invitrogen, Carlsbad, CA, USA) and chloroform. After extraction, RNA from the aqueous phase was precipitated using isopropyl alcohol, washed with 70% ethanol, dried and dissolved in deionized water and stored at −70°C. The integrity of the total RNA was evaluated by electrophoresis. Briefly, an aliquot of total RNA was subjected to electrophoresis on a 1% agarose gel with 1× TAE running buffer (Tris: Vetec, Rio de Janeiro, RJ, Brazil; EDTA: Sigma, St. Louis, MO, USA; acetic acid Vetec) and stained with Green™ Gel (Biotium, Hayward, CA, USA). The gel was analyzed using the software 1D LabImage (Kapelan Bio-Imaging Solutions, Leipzig, Germany) to confirm the absence of degraded genetic material. The concentration and purity of the total RNA samples was checked by determining the absorbance at 230, 260 and 280 nm in a spectrophotometer (UV-visible Ultrospec 3000, Pharmacia Biotech, Cambridge, England) [31]. Verification of the purity of the RNA samples was performed by assessing the presence of proteins with the 260/280 nm ratio, while contamination with aromatic compounds, such as phenol, was assessed with the ratio 260/230 nm. The benchmark used to evaluate these ratios (A260/A280 and A260/A230) was greater than or equal to 1.8, where A280 = the spectrophotometric absorbance at a wavelength of 280 nm and A230 = the spectrophotometric absorbance at a wavelength of 230 nm.

Total RNA was then precipitated using anhydrous sodium acetate at 3 mol/L and pH 5.2 (0.1 vol) (Sigma, St. Louis, MO, USA) and 100% ethanol (2.5 vol) at 4°C (Sigma, St. Louis, MO, USA, 2.5 times the volume 825 µL) and incubated at −20°C overnight. After incubation, the samples were centrifuged at 10000×*g* at 4°C for 30 min (Centrifuge Eppendorf 5415R, Hamburg, Germany). Ethanol (1 mL) (Sigma, St. Louis, MO, USA) was added to the pellet and it was centrifuged at 10000×*g* for 5 min at 4°C, dried at room temperature and diluted in 20 µL of deionized water. After this procedure, the absorbance at 230, 260 and 280 nm was determined (UV-visible Ultrospec 3000, Pharmacia Biotech, Cambridge, England) and the A260/A280 and A260/A230 ratios were again determined to assess the purity and concentration of the RNA in the treated material. Total RNA (1 µg) was used for cDNA synthesis reactions using a Reverse Transcription Kit Improm II System (Promega, Madison, WI, USA). Oligo (dT) primers were added to the total RNA and denaturation was performed at 70°C for 5 min. Improm-II Reverse Transcriptase was added and the samples were incubated at 42°C for 50 min, followed by inactivation at 70°C for 15 min. An attempt at cDNA synthesis without the reverse transcriptase enzyme was performed for each sample (negative control). An aliquot of the reaction control (no reverse transcriptase) was subjected to RT-PCR using a reaction system that had been tested and the oligonucleotide sequences had previously been used by other authors and described in scientific articles to confirm the absence of amplified genetic material.

### Determination of molecular biomarkers of aging and oxidative stress

The mRNA concentrations of the aging biomarkers ferritin light chain (Ftl), SMP30, hepcidin (Hamp), the transcription factor CREBh (Creb3l3), nuclear factor erythroid derived 2 like 2 (Nfe2l2), interleukin −1 beta (Il1b) and divalent metal transporter −1 (Slc11a2) were quantified using real-time polymerase chain reaction (qRT-PCR) (7500 Fast Real-Time PCR System, Applied Biosystems, Foster City, CA, USA). Real-time PCR was carried out using the Fast SYBR Green Master Mix 2X reagent (Applied Biosystems, Foster City, CA, USA) with 2 µL of cDNA (corresponding to 0.2 ng of total RNA) in a final volume of 10 µL with 5 µL Fast SYBR Green Master Mix and 0.2 µmol/L (final concentration) of each primer. The sequences of the primers used were designed by other authors: (1) ferritin light chain (Ftl), forward: CCTACCTCTCTCTGGGCTTCT; reverse: CTTCTCCTCGGCCAATTC [20]; (2) SMP30, forward: AGGCATCAAAGTGTCTGCTGTTT; reverse: GACTGTCGAAGTGCCACTGAACT [32]; (3) hepcidin (Hamp), forward: TGATGCTGAAGCGAAGGA; reverse: TGTGTTGAGAGGTCAGGAC [33]; (4) Creb3l3, forward: TCAGAGCCCTTTACCCAGACAG; reverse: ATGGTTGGGCTTAGGGTTCAG [22]; (5) nuclear factor erythroid derived 2 like 2 (Nfe2l2), forward: GAGACGGCCATGACTGAT; reverse: GTGAGGGGATCGATGAGTAA [34]; (6) interleukin-1 beta (Il1b), forward: CACCTCTCAAGCAGAGCACAG; reverse: GGGTTCCATGGTGAAGTCAAC [28]; (7) divalent metal transporter-1 (Slc11a2), forward: CTGATTTACAGTCTGGAGCAG; reverse: CACTTCAGCAAGGTGCAA; and (8) β-actin (Actb), forward: GTCGTACCACTGGCATTGTG; reverse: CTCTCAGCTGTGGTGGTGAA [35]. Dmt-1 primers can amplify iron-responsive element and non–iron-responsive element forms of Dmt-1 cDNA. Quantitative PCR was performed using a 7500 Fast Real-Time PCR System (Applied Biosystems, Foster City, CA, USA) for 40 cycles at 95°C for 20 s, 60°C for 3 s and 60°C for 20 s. The amplification specificity of each amplified product was verified using a melting curve. The efficiency of the qPCR amplification reactions was evaluated by running standard curves for each amplicon using different template dilutions of cDNA (1∶10, 1∶100, 1, 250, 1∶500, 1∶1000, 1∶2000 and 1∶10000). The expression of all genes was normalized to the expression of the housekeeping gene β-actin (Actb) and the reactions were run in duplicate. The amplification efficiency was determined from the slope obtained from the standard curve relating log[transcribed mRNA] and variation threshold cycle (C_T_) with the equation E = (10−1/slope-1)×100 and a slope value of the regression line plot of C_T_ values vs. log of input nucleic acid of approximately −3.32 was considered an efficient reaction. PCR efficiency was between 91 and 104% for all primers. A standard curve was plotted for each gene studied that correlated the ΔC_T_ (C_T_ target - C_T_ reference) versus the log of the cDNA amount and a slope value of the regression line plot of ΔC_T_ values vs. log of input nucleic acid of less than 0.1 was used as a general criterion to accept the validation of the experiment. From the data obtained previously, it had been determined to use a 1∶500 dilution of cDNA to analyze qRT-PCR where the efficiency obtained was higher than 99%. Melting curve analysis was used to examine the specificity of the products generated for each primer set. The comparative C_T_ method was used to quantitate the abundance of target gene mRNA and is given by 2^−ΔΔC^_T_. This method was performed as described in the tutorial “Guide to Performing Relative Quantitation of Gene Expression Using Real-Time Quantitative PCR” (Part #: 4371095 Rev B, Applied Biosystems).

### Immunoblot analysis

Rat livers were homogenized in four volumes of 0.25 M sucrose, 15 mM Tris-HCl (pH 7.9), 15 mM NaCl, 60 mM KCl, 5 mM EDTA, 0.15 mM spermine, 0.5 mM spermidine, 0.1 mM phenylmethanesulfonyl fluoride, 1.0 mM dithiothreitol and 1% protease inhibitor cocktail (Sigma Aldrich, St. Louis, MO, USA) and centrifuged at 12000×rpm for ten minutes. The supernatant was used as an extract [36]. Spot density of ferritin band was normalized to the amount of protein, by loading equivalent amount of protein (5 µg), in each lane, for each liver extract analyzed. Total protein concentration of standard ferritin [37] and animal's liver homogenates was determined by the method of Hartree et al. (1972) [38]. The ferritin light chain protein band has 19 kDa. The proteins were separated by 15% and 5% SDS-PAGE [31] and transferred to a 0.45 µm polyvinylidene difloride (PVDF) membrane (Immobilon®-P transfer membrane – IPVH00010 – Millipore – Billerica, MA, USA). And as a molecular weight standard protein was used in each gel and membrane (blot) a Spectra Multicolor Broad Range Protein Ladder (Thermo Scientific®) (Data not shown). Blots were first incubated for 1 h with blocking buffer (containing PBS, 0.1% Tween 20 and 5% nonfat dry milk) and then incubated with primary antibodies for 1 h at room temperature. Primary antibodies were used at a dilution of 1∶1000 for goat anti-FTL (SAB2500431 – Sigma Aldrich, St Louis, MO, USA). Blots were then washed three times in 0.1% PBS-T (Phosphate Buffered Saline with Tween 20) and incubated for 1 h at room temperature with 1∶3000 diluted anti-goat IgG alkaline phosphatase-conjugated secondary antibodies (A4187 - Sigma Aldrich, St Louis, MO, USA) and signals were visualized with BCIP®/NBT solution (B6404 – Sigma Aldrich, St Louis, MO, USA).

### Lipid peroxidation

The malondialdehyde (MDA) concentrations in liver, spleen, heart, skeletal muscle, gut and kidney homogenates were measured by high performance liquid chromatography (25 cm Shim-park C18 CLC-ODS (M) column Shimadzu, Kyoto, Japan – heart and kidney; and 15 cm Shim-park C18 CLC-ODS (M) column Shimadzu, Kyoto, Japan – liver, spleen, gut and skeletal muscle) [39]. The spectrofluorometric detector wavelengths were set at 532 nm (excitation) and 553 nm (emission). A four-point standard curve (0.81–16.16 nmol/mL) was made with tetraethoxypropane (TEP) (Sigma, St. Louis, MO, USA) dissolved in 1% H_2_SO_4_, as acid hydrolysis of TEP yields stoichiometric amounts of MDA (column 15 cm: y = 7.10^−7^× − 0.132; r^2^ = 0.9903 and column 25 cm: y = 7.10^−6^× + 0.0473; r^2^ = 0.9974). Total homogenate protein concentration was determined by the method of Hartree et al. (1972) [38]. The results were expressed as nmol MDA/mg total protein [38].

### Protein oxidation

Protein oxidation of liver, spleen, kidney, heart, gut and skeletal muscle homogenates was measured by carbonyl content according to the method of Richert et al. (2002) [40]. Absorbance was measured at 376 nm (spectrophotometer Shimadzu – TCC 240A) and carbonyl content was expressed as nmol of carbonyl groups per milligrams of total protein using an extinction coefficient of 22 mmol·L^−1^·cm^−1^. Total homogenate protein concentration was determined by the method of Hartree et al. (1972) [38].

### Preparation of tissue homogenates for enzyme assays

Liver, spleen, heart, gut, kidney and skeletal muscle tissues were homogenized at 1∶20 (w/v) in an Ultraturrax homogenizer using 0.5 mol/L of potassium phosphate buffer, pH 7.2, containing 50 mmol/L of ethylenediaminetetra-acetic acid and 1 mmol/L of phenylmethylsulfonyl fluoride (Sigma Aldrich, St. Louis, MO, USA) at 4°C and centrifuged at 15 000×*g* for 20 min.

### Catalase assay

The activity of catalase was quantified by the consumption of 10 mmol/L of H_2_O_2_ at 240 nm (spectrophotometer Shimadzu – TCC 240A) in buffer containing 10–50 µL of tissue homogenates [41], [42]. Blanks were run without H_2_O_2_. One unit of catalase was defined as the amount of enzyme required to decompose 1 µmol of H_2_O_2_/min.

### Glutathione peroxidase assay

Glutathione peroxidase activity was quantified using H_2_O_2_ as a substrate in a coupled assay with Glutathione Reductase-catalyzed oxidation of nicotinamide adenine dinucleotide phosphate (NADPH) at 340 nm (spectrophotometer Shimadzu - TCC 240A) [42]. First, the basal consumption of 0.15 mmol/L of nicotinamide adenine dinucleotide phosphate was measured in buffer containing 2 mmol/L of azide, 5 mmol/L of glutathione (GSH), 1.5 U of glutathione reductase and 10–50 µL of tissue homogenates. Then, 20 µL of H_2_O_2_ was added to a final concentration of 0.2 mmol/L. Blanks were run without tissue homogenate [43]. One unit of glutathione peroxidase was defined as the amount of enzyme required to oxidize 1 nmol of nicotinamide adenine dinucleotide phosphate/min.

### Glutathione Reductase assay

Glutathione reductase activity was quantified by spectrophotometry (spectrophotometer Shimadzu - TCC 240A) at 340 nm, where the oxidation of nicotinamide adenine dinucleotide phosphate was monitored for 20 seconds [42]. Tissue homogenate activity (75–250 µL) was measured in 50 mmol/L of potassium phosphate buffer (pH 7.2) containing 0.5 mmol/L of ethylenediaminetetra-acetic acid, 0.2 mmol/L of nicotinamide adenine dinucleotide phosphate and 1 mmol/L of glutathione oxidized (GSSG). Blanks were run only with GSSG. One unit of glutathione reductase was defined as the amount of enzyme required to oxidize 1 nmol of nicotinamide adenine dinucleotide phosphate/min.

### Glutathione-S-transferase assay

Glutathione-S-transferase (GST) activity was measured by following the conjugation of 1 mmol/L of GSH with 1 mmol/L of 1-chloro-2,4-dinitrobenzene [42] at 340 nm (spectrophotometer Shimadzu – TCC 240A) in 50 mmol/L of potassium phosphate buffer (pH 7.2) containing 50 µL of tissue homogenate [44]. One unit of GST was the amount of enzyme required to yield 1 nmol of product/min.

### NADPH oxidation assay

Nicotinamide adenine dinucleotide phosphate oxidase activity was measured by quantifying the consumption of NADPH by spectrophotometry (spectrophotometer Shimadzu - TCC 240A) at 340 nm [45]. The assay was monitored over 300 seconds and the assay buffer contained 50 mmol/L of potassium phosphate buffer (pH 7.2) with 0.5 mmol/L of ethylenediaminetetra-acetic acid, 0.1 mmol/L of nicotinamide adenine dinucleotide phosphate and 125–250 µL of tissue homogenate. One unit of NADPH oxidized was the amount of enzyme required to oxidize 1 nmol of NADPH/min.

### Statistical Analysis

Comparisons among the treatments in each group were tested by independent sample test t-test for equality of means using SPSS version 17 software (SPSS Inc., Chicago, USA). In all tests, a value of *P*<0.05 was considered statistically significant.

## Results

Table 1 shows the food and iron intake and the initial and final body weight of animals during the 78 days of treatment (12 weeks). There was no significant difference in food intake among the three groups of rats. As expected, ARI rats showed the lowest iron intake (p<0.001) and the AEI rats showed the highest intake of iron (p<0.001) compared to control rats. The adult rats from the three groups showed weight loss at the end of the treatment period relative to the initial time, however restricted-iron rats showed greater weight loss than control rats (p = 0.024).

**Table 1:** Food intake, iron intake, initial and final body weight of adult rats treated with control and restricted-iron (ARI) or enriched-iron (AEI) diets during a 78-day treatment period.

	Control	ARI	AEI
Food intake (kg)	1.83±0.04	1.68±0.16	1.79±0.09
Iron intake (mg)	64.22±1.13	16.81±1.60 (***)	627.64±34.29 (***)
Initial body weight (g)	526.52±42.45	510.75±79.97	514.62±39.38
Final body weight (g)	496.91±31.32	446.28±68.89	471.85±48.56

Average and standard deviation: n = 7 (CT); n = 5 (ARI); n = 8 (AEI). Difference between the control group and test groups:

* p≤0.05;

** p≤0.01;

*** p≤0.001 (Student's t-test).

The young group showed significantly higher hemoglobin concentrations (p = 0.006) and hematocrit levels (p = 0.001) than the control rats. Hematocrit levels were higher in the restricted-iron dietary group than in the control group (p = 0.036). The enriched-iron group showed higher hematocrit, serum iron and gamma glutamyl transferase levels (p = 0.025; p = 0.005 and p = 0.019, respectively) and lower C-reactive protein levels (p = 0.047) relative to the control group (Table 2).

**Table 2:** Hematological parameters of adult rats treated with control and restricted-iron (ARI) or enriched-iron (AEI) diets and of young rats.

	Young	Control	ARI	AEI
Erythrocytes (million/mm^3^)	8.82±0.34	8.62±0.48	8.90±0.68	8.75±0.39
Hemoglobin (g/dL)	17.3±0.9 (**)	15.8±0.5	16.5±0.6	16.2±0.8
Hematocrit (%)	51.6±3.2 (***)	45.1±1.0	47.4±1.7 (*)	46.7±1.2 (*)
Serum Iron (µg/dL)	242±106	226±34	164±99	346±78 (**)
Transferrin saturation (%)	58.9±22.3	63.7±19.8	39.6±23.6	75.2±13.2
Transferrin (mg/dL)	282±22	283±49	294±25	320±32
C-Reactive Protein (mg/dL)	0.33±0.11 (*)	0.48±0.06	0.56±0.12	0.38±0.11 (*)
Gamma glutamyl transferase (U/L)	3±2	1±0	1±0	7±5 (*)

Average and standard deviation: n = 7 (CT); n = 5 (ARI); n = 8 (AEI); n = 6 (young). Difference between the control group and test groups:

* p≤0.05;

** p≤0.01;

*** p≤0.001 (Student's t-test).

**Figure 1:**
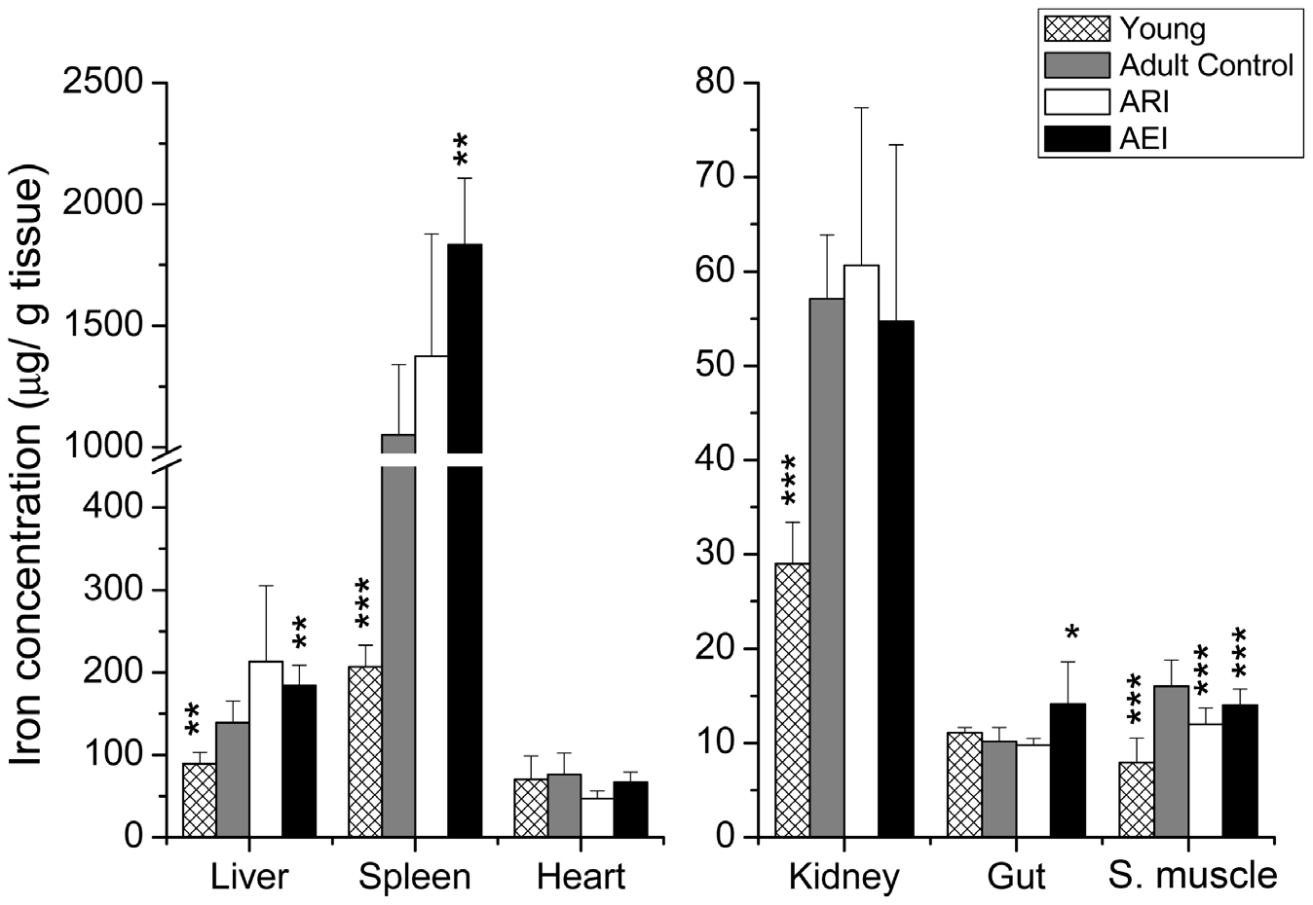
Concentration of iron in the tissues of adult rats treated with control and restricted-iron (ARI) or enriched-iron (AEI) diets and of young rats (µg of iron/g tissue). Average and standard deviation: n = 7 (CT); n = 5 (ARI); n = 8 (AEI); n = 6 (young). Difference between control group and test groups: *p≤0.05; **p≤0.01; ***p≤0.001 (Student's t-test).

The younger rats showed lower iron concentrations in liver, spleen, kidney and skeletal muscle relative to the control group (p = 0.001; p = 0.002; p<0.001 and p<0.001, respectively). Among the adult rats, the restricted-iron group only showed a reduction in iron concentration in skeletal muscle compared with the control group (p = 0.001) and no significant difference was observed in the iron concentrations of the other tissues. The livers, spleens and guts of the rats from the enriched-iron group showed higher iron concentrations (p = 0.005; p = 0.008 and p = 0.042, respectively), while in skeletal muscle this value was lower relative to the control group (p<0.001) (Figure 1).

**Figure 2:**
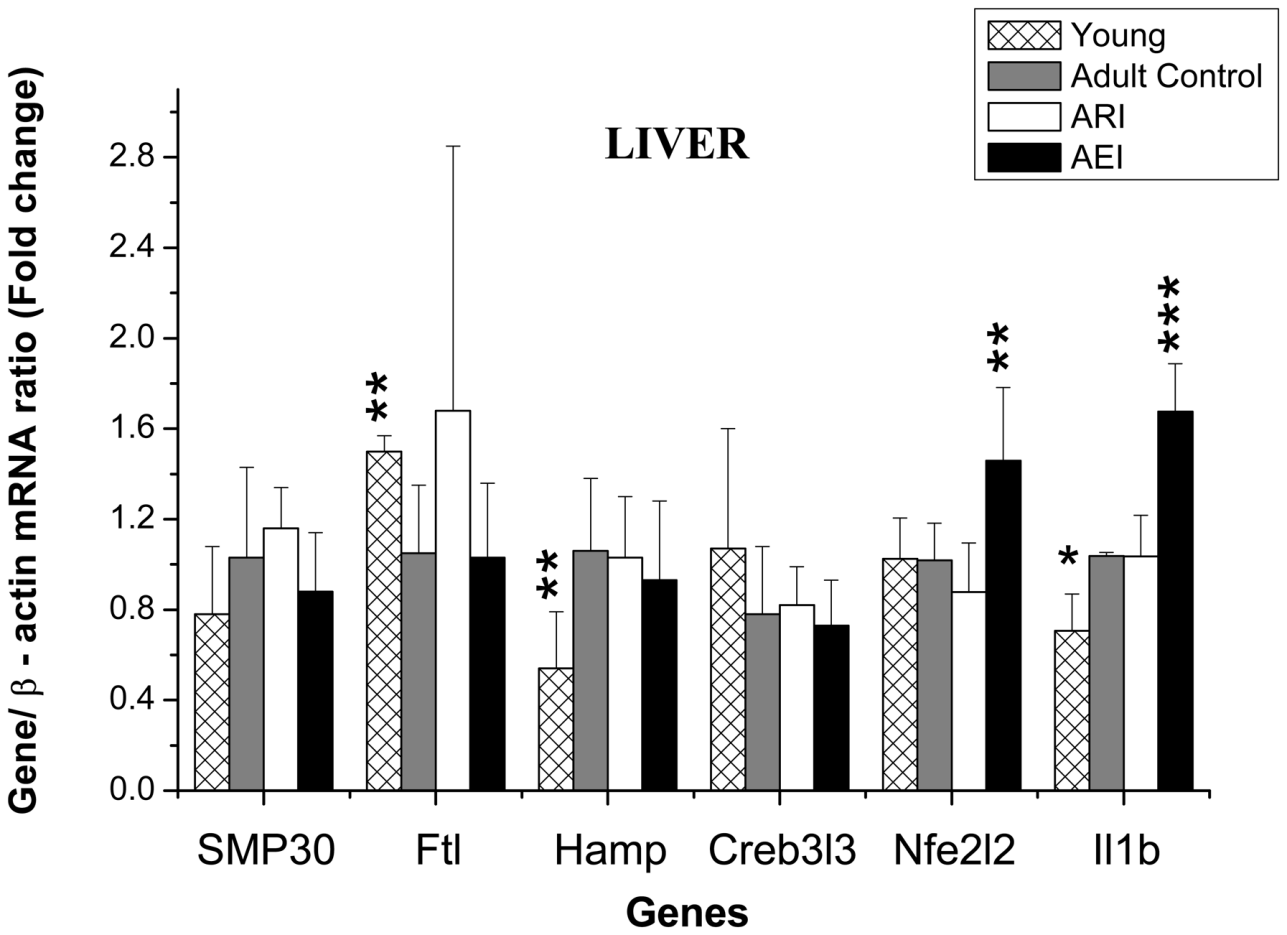
Quantification of SMP30, Ferritin (Ftl), Hepcidin (Hamp), Creb3l3, Nuclear factor erythroid derived 2 like 2 (Nfe2l2) and interleukin-1 beta (Il1b) mRNA from the livers of adult rats treated with control and restricted-iron (ARI) or enriched-iron (AEI) diets and of young rats (Gene/b-actin mRNA ratio - fold change). Average and standard deviation: n = 7 (CT); n = 5 (ARI); n = 8 (AEI); n = 6 (young). Difference between control group and test groups: *p≤0.05; **p≤0.01; ***p≤0.001 (Student's t-test). RT-PCR analyses were normalized against the mRNA expression of the housekeeping gene (β-actin gene) in the same tissue sample and then expressed as the “test/control” ratios.

Figure 2 shows the relative transcript levels of senescence marker protein 30 (SMP30), ferritin light chain (Ftl), hepcidin (Hamp), Creb3l3, nuclear factor erythroid derived 2 like 2 (Nfe2l2) and interleukin-1 beta (Il1b) in rat livers normalized to values obtained for β-actin (Actb). Young rats showed higher levels of hepatic ferritin light chain (Ftl) mRNA (p = 0.007), lower levels of hepcidin (Hamp) and interleukin-1 beta (Il1b) mRNAs (p = 0.008 and p = 0.004, respectively) compared to the control rats. There was no difference in the levels of hepatic SMP30 and Creb3l3 transcripts between the young and control groups. Higher transcript levels of Nfe2l2 and Il1b were found in the enriched-iron group relative to the control group (p = 0.009 and p = 0.001, respectively).

**Figure 3:**
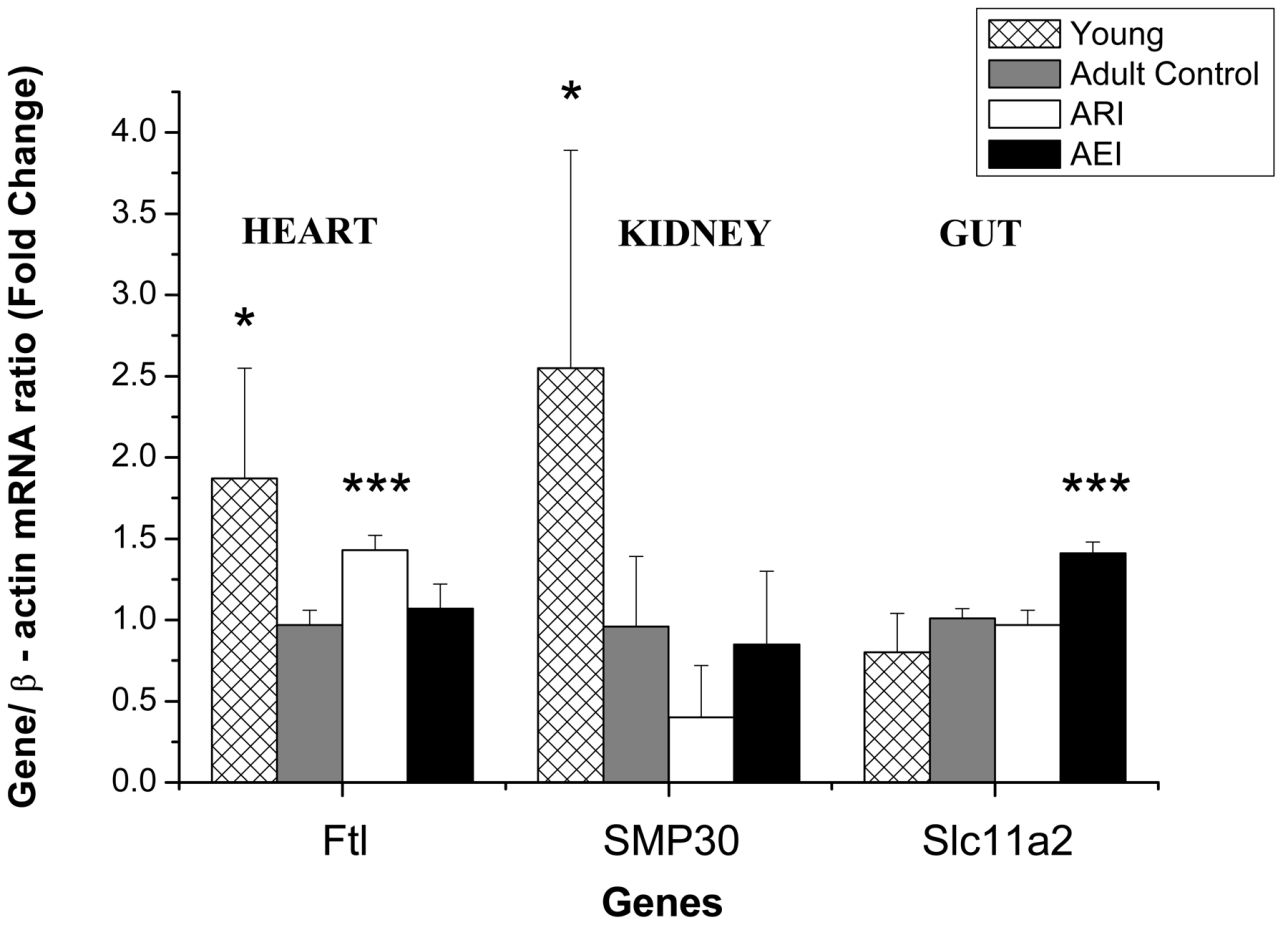
Quantification of Ferritin (Ftl) mRNA from heart tissue, SMP30 mRNA from kidney tissue and divalent metal transporter-1 (Slc11a2) mRNA from gut tissue of adult rats treated with control and restricted-iron (ARI) or enriched-iron (AEI) diets and of young rats (Gene/b-actin mRNA ratio - fold change). Average and standard deviation: n = 7 (CT); n = 5 (ARI); n = 8 (AEI); n = 6 (young). Difference between control group and test groups: *p≤0.05; **p≤0.01; ***p≤0.001 (Student's t-test). RT-PCR analyses were normalized against the mRNA expression of the housekeeping gene (β-actin gene) in the same tissue sample and then expressed as the “test/control” ratios.

The mRNA levels of Ftl, SMP30 and Slc11a2 were also analyzed in heart, kidney and gut, respectively, and the relative values are presented in Figure 3. The young group showed higher levels of kidney SMP30 transcripts and higher heart Ftl transcripts relative to the control group (p = 0.033 and 0.022, respectively). Among the adult groups, ARI rats showed higher levels of heart Ftl mRNA and a marginal reduction in the levels of kidney SMP30 mRNA relative to the control group (p<0.001 and 0.060, respectively). The AEI rats showed higher levels of gut Slc11a2 mRNA than the control group (p<0.001). There was no difference in heart Ftl and kidney SMP30 mRNA levels between the AEI and control groups (Figure 3).

**Figure 4:**
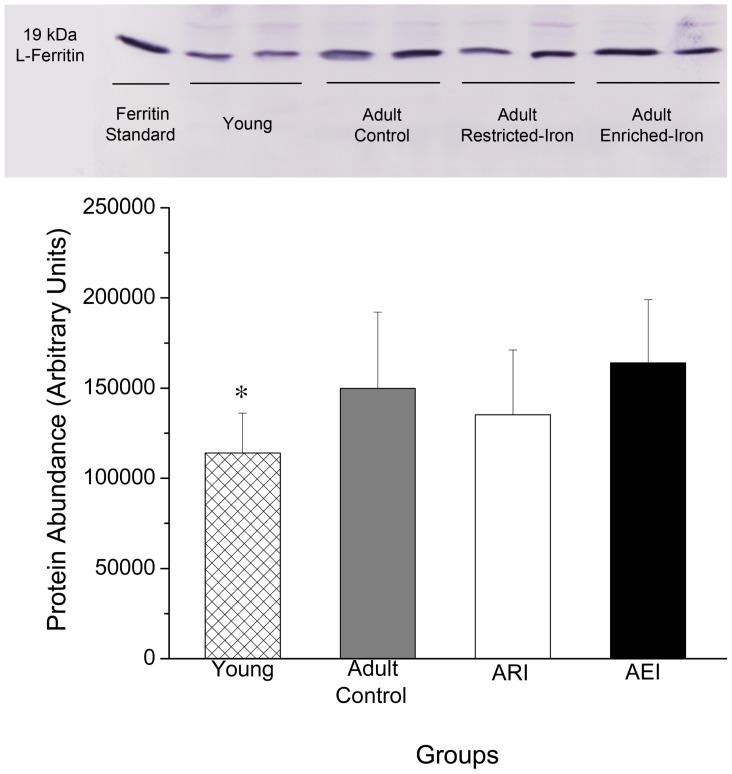
Western Blot analysis of Ferritin Light Chain (19 kDa) protein expression in liver (5 µg) from adult rats treated with control and restricted-iron (ARI) or enriched-iron (AEI) diets and in young rats. Average and standard deviation: n = 7 (CT); n = 5 (ARI); n = 8 (AEI); n = 6 (young). Difference between control group and test groups: *p≤0.05; **p≤0.01; ***p≤0.001 (Student's t-test).

Ferritin light chain protein levels were analyzed by Western blot in the livers of rats from all groups and the results are shown in Figure 4. A significant difference was only found for young rats, which showed lower levels of ferritin protein compared to the control group (p = 0.046).

**Figure 5:**
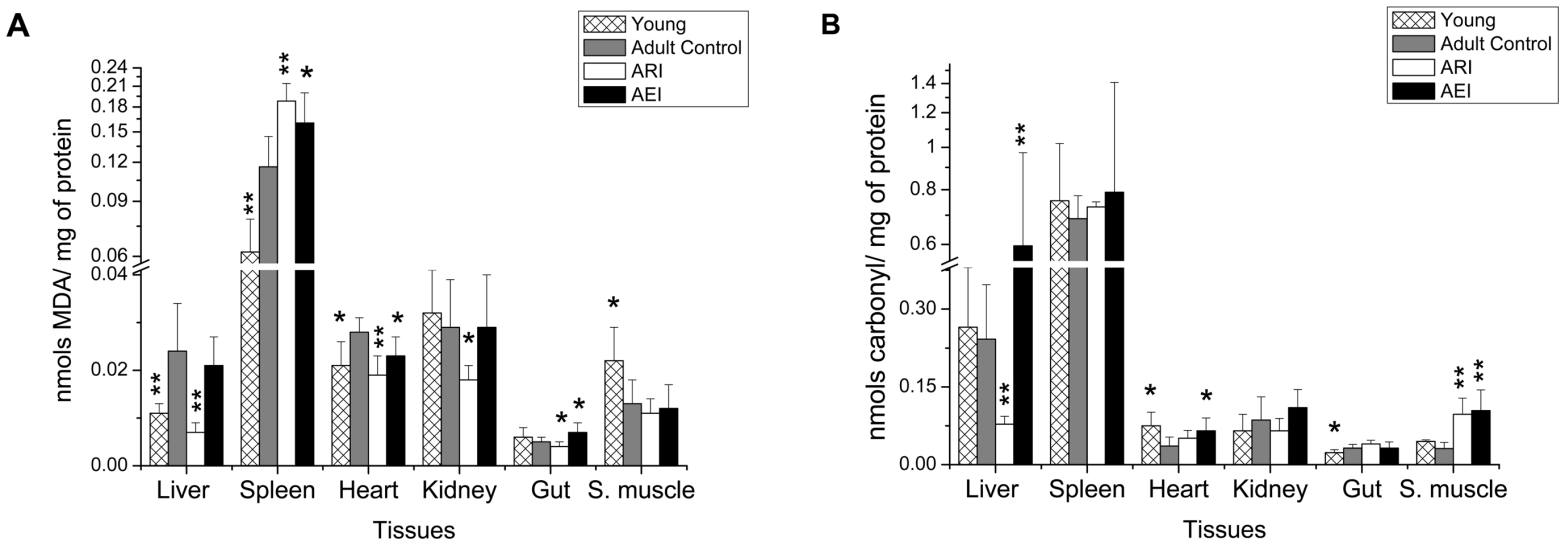
Malondialdehyde (MDA) (A) and protein carbonyl concentration (B) in liver, spleen, heart, kidney, gut and skeletal muscle homogenates of young rats and adult rats treated with control and restricted-iron (ARI) or enriched-iron (AEI) diets (nmols MDA/mg of protein and nmols carbonyl/mg of protein). Average and standard deviation: n = 7 (CT); n = 5 (ARI); n = 8 (AEI); n = 6 (young). Difference between control group and test groups:*p≤0.05; **p≤0.01; ***p≤0.001 (Student's t-test).

Figure 5 shows the oxidative state of the rats, which was evaluated by measuring carbonyl and MDA levels in rat tissues. The young group showed significantly lower levels of MDA in liver, spleen and heart and higher levels in skeletal muscle relative to the control group. Young rats also showed lower carbonyl levels in gut; however, higher levels were found in the heart. Restricted-iron diet rats showed a significant reduction in MDA levels in liver, heart, kidney and gut and a significant increase in MDA levels in spleen relative to control rats. Carbonyl levels were also significantly lower in the livers of ARI rats than control rats, but increased carbonyl levels were observed in skeletal muscle. The enriched-iron (AEI) rats showed a significant increase in MDA levels in spleen and gut and a significant reduction in heart tissue. Liver, heart and skeletal muscle of the AEI rats showed higher carbonyl levels than control rats (Figure 5).

**Figure 6:**
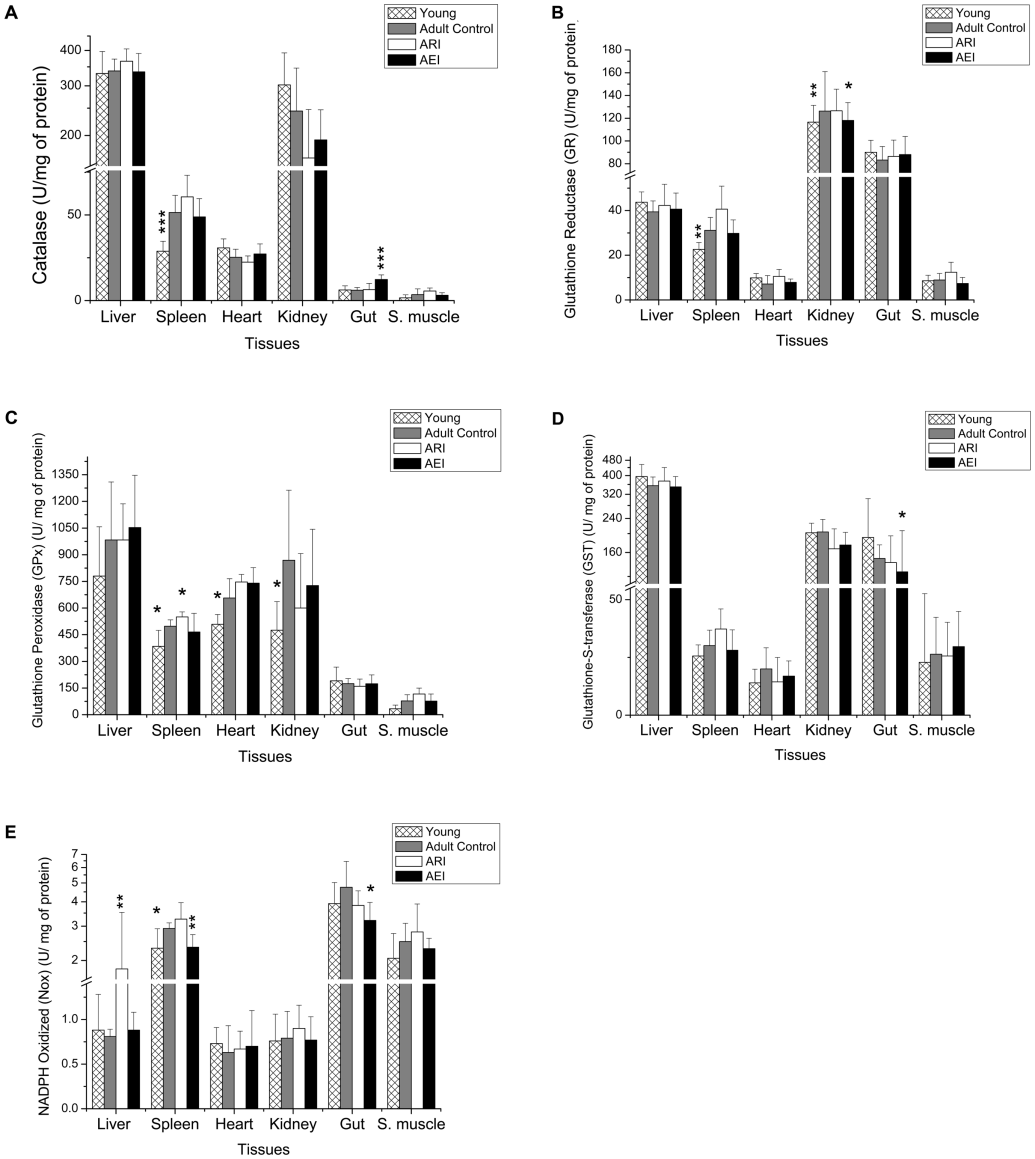
Specific activity of catalase (A), GR (B), GPX (C), GST (D) and Nox (E) from tissue homogenates of adult rats treated with control and restricted-iron (ARI) or enriched-iron (AEI) diets and of young rats (U/mg of protein). Average and standard deviation: n = 7 (CT); n = 5 (ARI); n = 8 (AEI); n = 6 (young). Difference between control group and test groups:*p≤0.05; **p≤0.01; ***p≤0.001 (Student's t-test). GR, glutathione reductase; GPX, glutathione peroxidase; GST, glutathione-S-transferase; Nox, Nicotinamide adenine dinucleotide phosphate oxidase.

The oxidative status of young and adult rats was also measured by analyzing the specific enzymatic activities of catalase (CAT), glutathione reductase (GR), glutathione peroxidase (GPx), glutathione S-transferase (GST) and NADPH oxidase (Nox) and the results are shown in Figure 6. There were changes in specific enzymatic activities in some tissues. Catalase activity was higher in liver and kidney, GPx activity was higher in liver, spleen, heart and kidney, GR activity was higher in kidney and gut, GST activity was higher in liver, kidney and gut and Nox activity was higher in spleen, gut and skeletal muscle. Young rats showed significantly lower levels of CAT activity in spleen, while GR activity was lower in spleen and kidney, GPx activity was lower in spleen, heart and kidney and Nox activity was lower in spleen relative to control rats. The ARI rats only showed differences in the activity of two antioxidant enzymes: GPx in spleen and Nox in liver were higher than the levels found in control rats. AEI rats showed high levels of CAT activity in gut and there was lower activity for GR in kidney, GST in gut and Nox in spleen and gut compared to control rats (Figure 6).

## Discussion

Oxidative stress and aging are related to the decline in the repair mechanisms for molecular oxidative damage, which can cause cellular dysfunction, compromising organ function and, consequently, organism life span [13]. Free iron in the body generates oxidative stress, and, therefore, an excess of iron in the body might accelerate the aging process [13], [46]. To test this hypothesis in the present study, adult Wistar rats (15 months of age) were treated with diets containing adequate levels of iron (adult control group), restricted-iron levels (ARI group) and enriched-iron levels (AEI group) to verify the effects of dietary iron content on iron status, oxidative stress and on aging markers in rat tissues. Wistar rats aged two months constituted a young control group for comparison with the data obtained from adult rats.

Tissue iron accumulation in senescent animals and humans seems to be a result of an increase in inflammatory processes during the aging [12], [17], [18], [47], [48]. Actually, the cytokines IL1β and IL6 induce hepatic synthesis of hepcidin [10], an antimicrobial hormone, which, once secreted into the bloodstream [49], [50], reduces intestinal iron absorption and prevents iron exportation from macrophages of the spleen to the bloodstream [8], [10]. Thus, high levels of hepcidin impair iron mobilization, leading to iron accumulation in tissues such as the spleen and liver [33], [51]. Iron overload in the tissues of patients with chronic diseases such as renal failure, or diseases of aging, such as Parkinson's [52], supports the hypothesis that excess iron in an organism is involved in the pathology of the aging process. Moreover, reduced iron mobilization by the hormone hepcidin impairs several physiological processes that are iron-dependent, such as erythropoiesis [49], which may lead to anemia.

In the present study, rats at 15 months of age were considered adult rats, while rats at 2 months of age were considered young. However, the weight loss observed during the 78-day treatment, as well as the reductions in hemoglobin concentrations and hematocrit values found in rats from the 15-month control group compared to young rats, are characteristic of the senile stage of the first group (Table 1 and 2) according to the results reported by Arvapalli et al. (2010) [17].

The adult rats of the control group showed higher values of serum C-reactive protein (CRP) (Table 2) and higher levels of hepcidin transcripts (Figure 2) associated with increased iron in liver, spleen, kidney and skeletal muscle (Figure 1) and they also showed lower hemoglobin and hematocrit levels (Table 2) relative to young rats. Moreover, adult rats had higher level of interleukin-1 beta mRNA (Il1b) (Figure 2), a pro-inflammatory cytokine that up regulates liver hepcidin [53]. An activation of the innate immune system and the presence of low-grade inflammation is a common hallmark of the aging process [54]. Put all together, these results corroborate with the hypothesis that the induction of hepcidin transcription could be responsible for the reduction in iron mobilization in adult rats, resulting in iron tissue accumulation and consequently, a reduction of erythropoiesis.

In accordance with the hypothesis that oxidative stress is associated with the aging process [55], young rats had lower levels of oxidative damage to lipids in the liver, spleen and heart compared to adult control rats. The higher levels of oxidative damage to heart proteins and skeletal muscle lipids found in the young rats (Figure 5) that were apparently contrary to the Harman hypothesis, could be explained by the high metabolic and physical activities of the young rats compared to the adult rats.

The lower specific activities of the antioxidant enzymes CAT, GR and GPx and the Nox enzyme that were observed in some tissues of young rats compared to adult rats (Figure 6) is in agreement with findings in the literature. Researchers observed an increase in GPx activity in the livers of aged male mice [56]. Some authors have found increased CAT and GPx activity during the first 18 months of life in rats and mice, but these values decreased significantly at 24 months of age [56], [57]. Similar to results from rat spleens, shown in Figure 6, reduced levels of Nox in young rats have been reported in the literature [58], [59], [60]. Together, these results suggest that levels of the specific activities of antioxidant enzymes tend to increase with the age.

The role of dietary iron in the aging process is not fully explored. The deleterious effects of dietary iron seem not to be due to high iron intake but rather to abnormal cellular iron metabolism, trafficking or storage [61]. Surprisingly, adult rats submitted to the restricted-iron diet (ARI) after 12 weeks of treatment showed a significant reduction in iron content only in skeletal muscle compared to the control group. This result suggests that the iron that accumulates in tissues with age can be found predominantly in the hemosiderin complex in an iron redox-inactive form [13], which protects tissues against the oxidative stress induced by iron [62]; however, this iron form is not bioavailable for physiological functions. If so, it is expected that a chronic reduction in dietary iron would induce anemia in rats. The ARI rats did not show anemia, but instead they showed higher hematocrit levels than the control group. These results may be due to the effect of hemoconcentration because these animals lost more weight compared to control rats.

Although a reduction in iron concentration was observed only in skeletal muscle, the consumption of a restricted-iron diet contributed to the significant reduction in oxidative damage to lipids (MDA) in liver, heart, kidney and gut and a reduction in carbonyl levels in the livers of ARI rats. A similar result was obtained in a recent study conducted in young rats, in which it was observed that dietary restriction of iron reduced the production of superoxide and lipid peroxide levels in the liver, pancreas and plasma [63]. Thus, except for the increased oxidative damage to lipids and proteins observed in the spleen and muscle, respectively, the data obtained from the other organs of the rats with the restricted-iron diet correlates with evidence reported in the literature and corroborates the thesis that iron deprivation or dietary iron chelation therapy may be effective in treating or preventing oxidative stress and thus retard the aging process [18]. However, one must consider that iron accumulated in the tissues of the elderly may be predominantly stored in hemosiderin complexes and is therefore unavailable to meet physiological demands. A marginal reduction was observed in the transferrin saturation values of the ARI rats compared to control rats (p = 0.0840), suggesting a reduction in iron mobilization in these rats. Thus, both iron restriction and the use of chelating agents may reduce the amount of bioavailable iron, which could impair several cellular processes and tissue functions. The reduced iron levels associated with higher body weight loss (Figure 1, Table 1) and the increased oxidative damage to proteins (Figures 1 and 5 and Table 1) observed in the skeletal muscle of ARI rats suggest a more pronounced loss of muscle, most likely due to the depletion of bioavailable iron in these animals.

In the other hand, the increased values of hematocrit, serum iron and iron concentration in liver, spleen and gut (Table 2 and Figure 1) found in the adult enriched-iron group, indicate an increased absorption of dietary non-heme iron in these rats. These results, in association with increased oxidative stress (Figure 5), as evidenced by increased serum levels of gamma glutamyl transferase (Table 2), MDA in spleen and gut (Figure 5) and carbonyl in liver, heart and skeletal muscle (Figure 5), suggest that dietary iron supplementation leads to tissue iron overload and also increase oxidative stress in tissues, despite the thin molecular mechanism of gut iron absorption regulation that is synchronized with physiological demands [8].

Ferritin levels in tissues indeed increase during the aging process [62], [64]. In mammals, most non-heme iron is linked to ferritin, a molecule that consists of two types of polypeptide chain, light (L) and heavy (H), whose function is to reduce the intracellular labile iron pool, minimizing production of ROS [21] and, therefore, protecting cellular components against oxidative damage [11], [62], [64], [65]. The expression of ferritin in tissues is translationally regulated by labile intracellular iron through the IRP/IRE system (for review see [11], [66]).

As expected, Western blot results showed that young rats had lower levels of hepatic L-ferritin protein (Figure 4), while the mRNA levels of L-ferritin (Ftl) in the livers and hearts of young rats were significantly higher compared to adult rats of the control group (Figure 2 and 3). A similar result was also observed in the livers of young rats (2 months) compared to aged rats (24 months) in a study by Bulvik et al. (2012) [62]. These results, suggest that, in addition to the major role of ferritin in the reduction of intracellular labile iron, the L-ferritin mRNA molecule is also stabilized by the IRP/IRE system at low intracellular iron concentrations. Specifically, although cells with low iron content do not synthesize L-ferritin protein due to the binding of IRP to 5′ IREs, L-ferritin mRNA molecules should be maintained intracellularly to allow the immediate translation of ferritin. This strategy would quickly prevent increases in cellular oxidative stress when cells are exposed to high concentrations of iron. The increase of the heart Ftl mRNA levels found for the adult restricted-iron rats (Figure 3) corroborate with this thesis.

In higher animals, hepatic levels of SMP30 protein decrease during the aging process [21], [67], [68], [69]. SMP30 is a protein that is predominantly synthesized by the liver and kidneys with highly conserved amino acid sequences among various animal species, including humans. Although its genuine function in humans is still unknown, studies in a human hepatoma cell line suggest that SMP30 regulates the levels of cytoplasmic Ca^2+^ through the modulation of sodium-independent Ca^2+^-pumping activity in plasma membranes. In rats, this protein participates in the synthesis of vitamin C [68] and appears to protect cells against apoptosis and other lesions [70], moreover there is a negative correlation between the expression of SMP30 and levels of reactive oxygen species in the liver and kidneys of mice [69]. Thus, the reduction of SMP30 with aging appears to be associated for the senescent fragility that contributes to the deterioration of cellular functions. In agreement with the literature, young rats showed higher levels of SMP30 transcripts in the kidney compared to adult rats. Interestingly, rats treated with the restricted-iron diet, which, despite the reduction in oxidative damage to lipids, showed a marginal reduction (p = 0.060) in kidney SMP30 levels compared to control rats.

Cells have several molecular defense mechanisms against oxidative stress. CREBh, a hepatocyte-specific transcription factor belonging to the cyclic AMP response element binding protein transcription factor (CREB/ATF) family [71], [72] is activated by endoplasmic reticulum stress. Once activated, CREBh regulates iron metabolism through the induction of hepcidin [50], and it is also required to induce the expression of serum amyloid P-component and C-reactive protein, which are both involved in the acute phase response [73]. The oxidative stress are also regulated by the transcription nuclear factor erythroid derived 2 like 2 (Nfe2l2) in multiple cell types and tissues, including the liver [25], [26]. Nfe2l2 controls the basal and induced expression of an array of antioxidant responsive element-dependent genes that regulate the physiological and pathophysiological outcomes of oxidant exposure [25]. In the present study, although there were no changes in the levels of hepatic Creb3l3 and Nfe2l2 mRNAs, young rats showed lower hepatic oxidative stress and also lower serum CRP and liver mRNA Hamp levels in comparison to adult rats.

The result regarding the transcript factor nuclear factor erythroid derived 2 like 2 (Nfe2l2) found in the liver of iron supplemented rats associated with the high levels of hepatic iron strengthens the previous findings that elevated hepatic iron levels induce oxidative stress [26] once these animals showed the higher transcript levels of Nfe2l2 and higher iron concentration relative to the control group (Figure 2). In the other hand, the highest value of liver Il1b mRNA level also found in the enriched-iron rats suggest an enhanced of inflammatory process. These results support the role of hepatic oxidative stress in the acute phase response and iron metabolism and also indicate a link between aging, oxidative stress and inflammatory processes. The apparently contradictory result found in the enriched-iron rats, which showed lower CRP levels than the control rats (p = 0.047), may, actually, reflects a liver dysfunction as a consequence of the increase in oxidative stress, as evidenced by the increased of carbonyl protein in the liver of these rats (Figure 5B).

In regard to the systemic iron regulation the young rats showed lower Hamp mRNA levels relative to the control group, however there was no significant difference among the three adult groups. This finding should be due to the putative saturable physiological response of the hepcidin recently postulated by Daba et al. (2013) [74]. The high iron concentration found in the adult rats, may be higher than the hepcidin saturation response. Thus it may not respond any more to serum and liver iron concentration changes, resulting in the impairment of the systemic iron homeostasis. The saturable hepcidin response may explain the changes in iron tissues and in the oxidative status of the adult rats, which were proportional to the amount of iron in the diets. Considering the recently findings that hepcidin inhibits the expression of Dmt1 [75] we also analyzed the Dmt1 transcripts levels (Slc11a2) in the gut of the rats (Figure 3). Surprisingly, although the mRNA level of hepcidin did not change among the adult groups, the enriched-iron rats showed higher transcript levels of Slc11a2 (Dmt1) than control group. This result suggests another systemic regulatory mechanism of iron absorption hepcidin-independent. A recent study showed that the proinflammatory cytokine IL-1β induces divalent metal transporter 1 (Dmt1) expression [76]. In the present study, the supplemented rats showed also higher levels of mRNA for Il1b. Taken together, it is possible that increased levels of Slc11a2 (Dmt1) are due to inflammatory process.

## Conclusion

Despite the molecular mechanisms for the regulation of iron absorption, the chronic consumption of a diet with high iron content by adult rats resulted in the accumulation of iron in the liver, spleen and gut, increased oxidative stress in the majority of studied tissues and reduced the activity of some antioxidant enzymes. Although the restriction of dietary iron only reduced iron levels in skeletal muscle, with the exception of spleen and muscle, it protected the other studied tissues against oxidative damage to lipids and proteins and resulted in increased activity of GPx in spleen. However, dietary restriction of iron also caused higher weight loss, increased oxidative stress in muscle and spleen and resulted in high activity of Nox in liver of adult rats. The results of this study indicated that iron supplementation may accelerate aging processes by increasing tissues oxidative damages, while iron restriction may retard. However, iron restriction may also impair other physiological processes that are not associated with aging. Considering the limitations of the animal model used in this study, the treatment of elderly patients with iron chelators could reduce oxidative stress in some tissues but could also lead to cell dysfunction due to the depletion of bioavailable iron. On the other hand, the iron fortification of several foods, which occurs in various countries, may increase oxidative stress and, consequently, compromise both the quality and the life expectancy meanly of elderly populations. In conclusion, adult organism may be more vulnerable to changes in the concentrations of dietary iron than young, because they lose their capacity to control the iron absorption by the modulation of hepcidin levels.
